# Case Report: Papillary renal cell carcinoma complicated by ipsilateral renal tuberculosis

**DOI:** 10.3389/fmed.2025.1597849

**Published:** 2025-08-12

**Authors:** Zhong Tian, Cheng Zhu, Tingting Yang, Shicheng Chen, Zhongcong He, Guang Han, Zhouhui Chen, Neng Zhang, Bo Yu, Ni Fu

**Affiliations:** ^1^Department of Urology, The Second Affiliated Hospital of Zunyi Medical University, Zunyi, China; ^2^Department of Urology, The Affiliated Hospital of Zunyi Medical University, Zunyi, China

**Keywords:** renal cell carcinoma, renal tuberculosis, tumor, papillary renal cell carcinoma, renal cell carcinoma with renal tuberculosis

## Abstract

Papillary renal cell carcinoma (PRCC) complicated by ipsilateral renal tuberculosis (TB) represents an exceptionally rare and complex clinical condition. Renal TB is the most common form of urogenital TB, while PRCC is the most prevalent histological subtype of non-clear cell renal cell carcinoma (RCC). In this study, we present the first reported case of PRCC complicated by ipsilateral renal TB, where the patient exhibited low back pain without hematuria. Initial imaging studies indicated a space-occupying lesion in the left kidney, raising suspicion of renal tumors. Subsequent postoperative pathology, immunohistochemical staining, and tuberculosis PCR results confirmed the diagnosis of PRCC complicated by ipsilateral renal TB.

## Introduction

RCC is the most common histological subtype of renal malignancies (1) and is also considered one of the most lethal forms of urinary tract cancer (2, 3). While the incidence of RCC has increased in recent years, its mortality rate has decreased (4, 5). Possible reasons for this trend include the increased detection rate of renal cancer through abdominal CT scans and advancements in systemic treatment regimens (6). Genitourinary TB is the second most common form of extrapulmonary TB, with renal TB being the most prevalent manifestation (7). Ipsilateral occurrence of RCC and renal TB is extremely rare. In this report, we present a clinical case that illustrates this uncommon coexistence.

The patient initially sought medical attention due to unexplained lower back pain. Based on imaging findings, the initial diagnoses included a space-occupying lesion in the left kidney, a left kidney stone, and left kidney atrophy. Consequently, laparoscopic radical resection of the left renal tumor was performed. Postoperative pathology revealed neoplastic lesions with caseous necrosis, leading to the diagnosis of RCC coexisting with renal TB. The final diagnosis of ipsilateral PRCC coexisting with renal TB was confirmed through immunohistochemical staining and tuberculosis PCR.

## Case report

**Figure 1:**
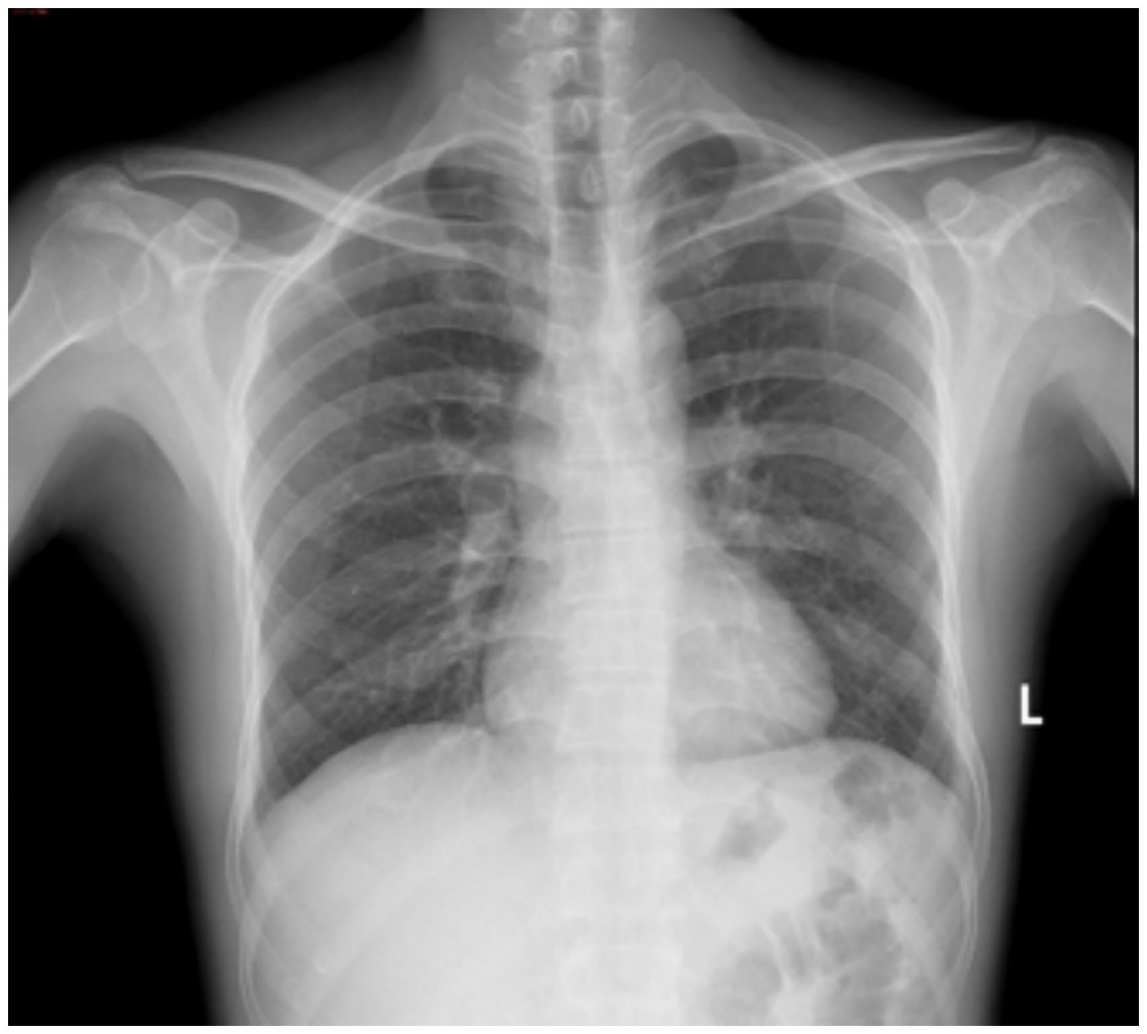
The chest X-ray revealed no significant abnormalities.

**Figure 2:**
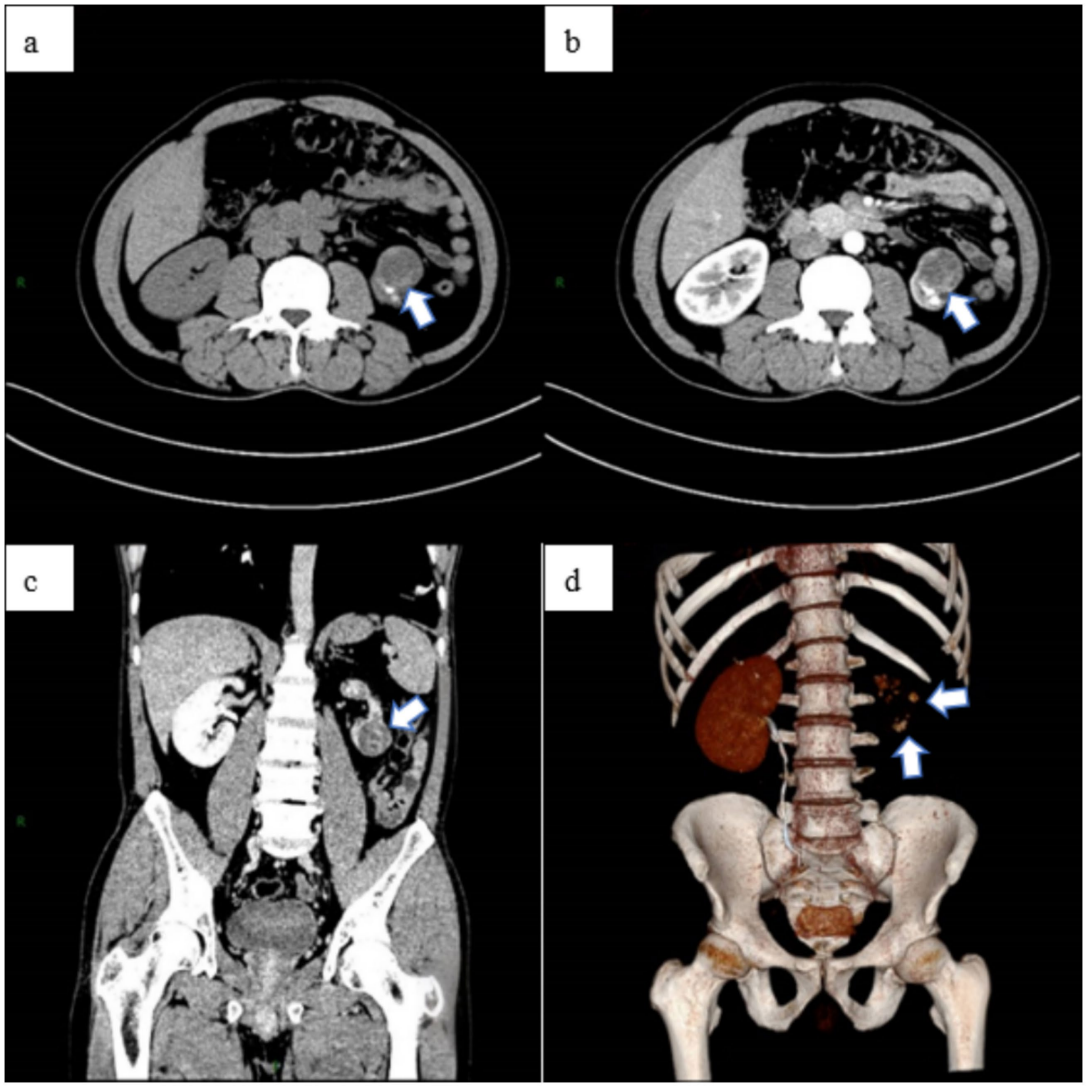
The abdominal CT scan revealed a space-occupying lesion in the left kidney, measuring approximately 28 × 29 × 31 mm **(a)**, along with multiple stones. The abdominal enhanced CT showed uneven enhancement of the lesion **(b)**, located in the lower pole of the left kidney **(c)**, and a reduction in the volume of the left kidney. Additionally, the left kidney and ureter were not visualized, indicating non-functionality of the left kidney **(d)**.

A 51-year-old male patient was initially evaluated at another hospital 2 years ago for lumbar pain, during which left renal atrophy was identified; however, no further treatment was administered. Recently, he presented with recurrent low back pain lasting for 1 month, without an identifiable cause, and without accompanying hematuria or symptoms of bladder irritation. Despite the symptoms, the patient has maintained stable mental health and healthy dietary habits. Subsequent examinations at other hospitals revealed a left renal space-occupying lesion and continued renal atrophy. For comprehensive diagnosis and treatment, the patient was referred to our hospital. A chest X-ray (Figure 1) revealed no evidence of disseminated or cavitary pulmonary TB. Abdominal CT imaging revealed a space-occupying lesion measuring approximately 28 × 29 × 31 mm in the left kidney, along with multiple kidney stones and reduced kidney volume (Figure 2), suggesting the presence of a potential tumor. An MRI examination was recommended for further evaluation. The patient was subsequently admitted for additional assessment and treatment.

**Figure 3:**
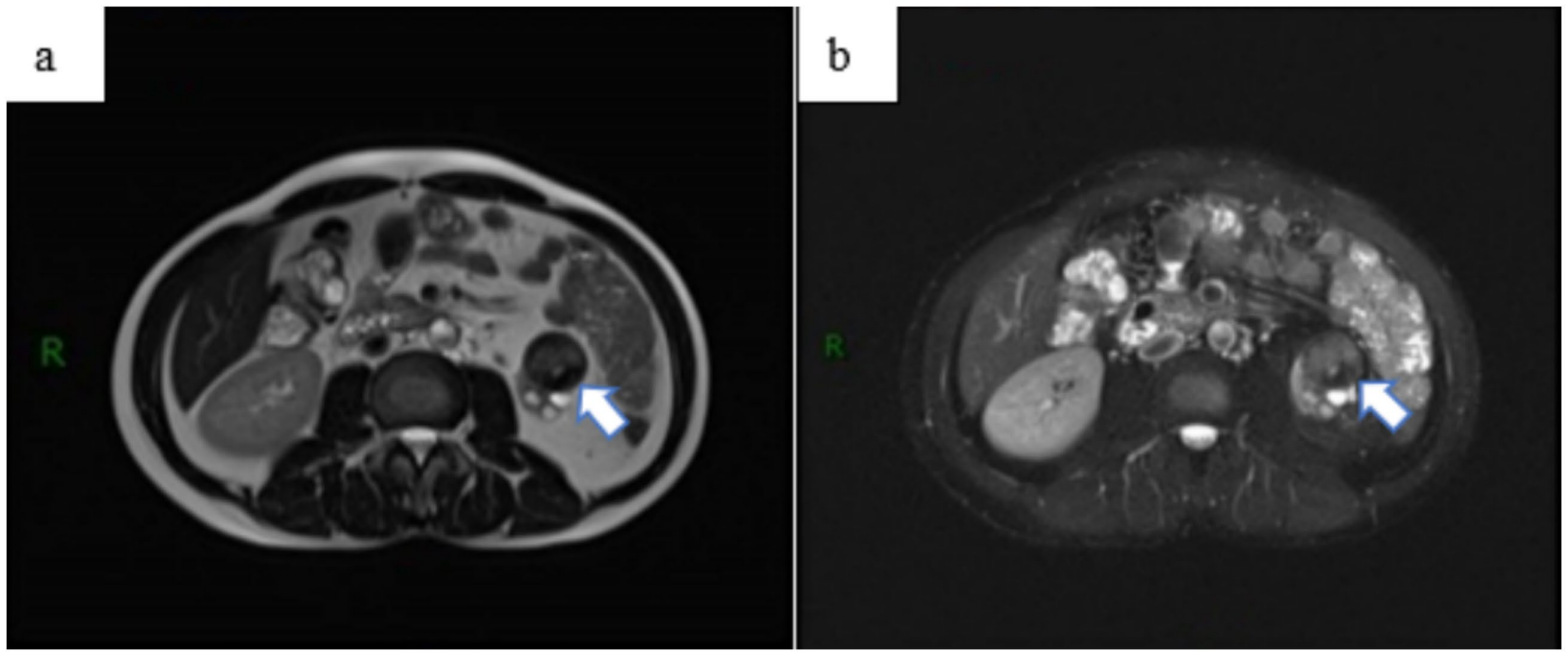
Abdominal MRI examination revealed a space-occupying lesion outside the renal contour of the left kidney, **(a)** with no significant enhancement observed on the enhanced scan **(b)**. The lesion was classified as a renal tumor, stage T3aN0M0.

Upon admission, the patient reported no prior history of surgery, infectious diseases, or chronic conditions. Physical examination revealed no palpable abdominal masses, no percussion tenderness over the bilateral kidneys, and no significant findings in other diagnostic tests. The laboratory test results suggest that the patient may have been previously infected with *Mycobacterium tuberculosis (MTB)* or could currently be in the latent phase of infection (Table 1). Abdominal MRI indicated a heterogeneous signal mass measuring approximately 28 × 31 × 31 mm at the lower pole of the left kidney (Figure 3), suggestive of a renal tumor with multiple bilateral renal cysts. The initial diagnosis included a left kidney tumor, multiple kidney stones in the left kidney, and atrophy of the left kidney.

**Table 1:** Laboratory studies.

Laboratory study	Result	Laboratory study	Result
Complete blood test		Prealbumin	188
Hemoglobin, g/L	128	A/G	1.6
Total leukocyte count, × 10^9^/L	8.78	Urinalysis	
Neutrophil count	5.97	pH	6.0
Lymphocyte count	1.49	Specific gravity Proteinuria	1.020
Platelet count, × 10^3^/L	140	Ery	Negative
MTB nucleic acid testing (sputum)	Negative	U-LEU	Negative
T-SPOT (blood)	Positive	Urinalysis	Negative
Blood urea nitrogen, mg/dL	8.4	GLU	Negative
Serum creatinine, mg/dL	1.17	Nitrite	Negative
AST, U/L	24	Anti-HIV antibody	Negative
ALT, U/L	16	Syphilis antibody	Negative
Blood albumin, g/L	30.9	HBs antigen	Negative
Globulin, g/L	19.3	Anti-HCV antibody	Negative

**Figure 4:**
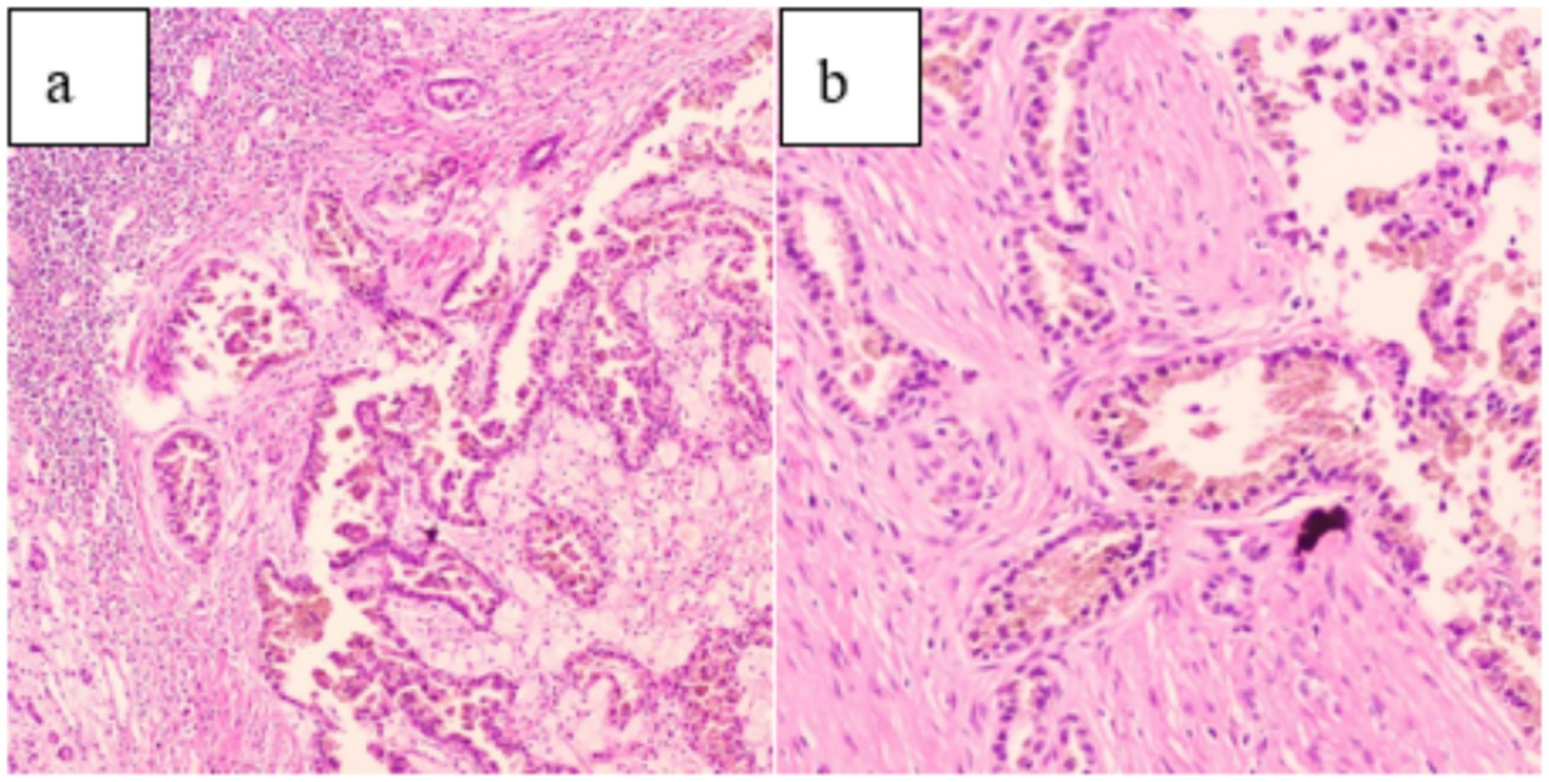
**(a)** (HE 10 × 10) and **(b)** (HE 20 × 10) demonstrate tumor cell infiltration on pathological examination.

**Figure 5:**
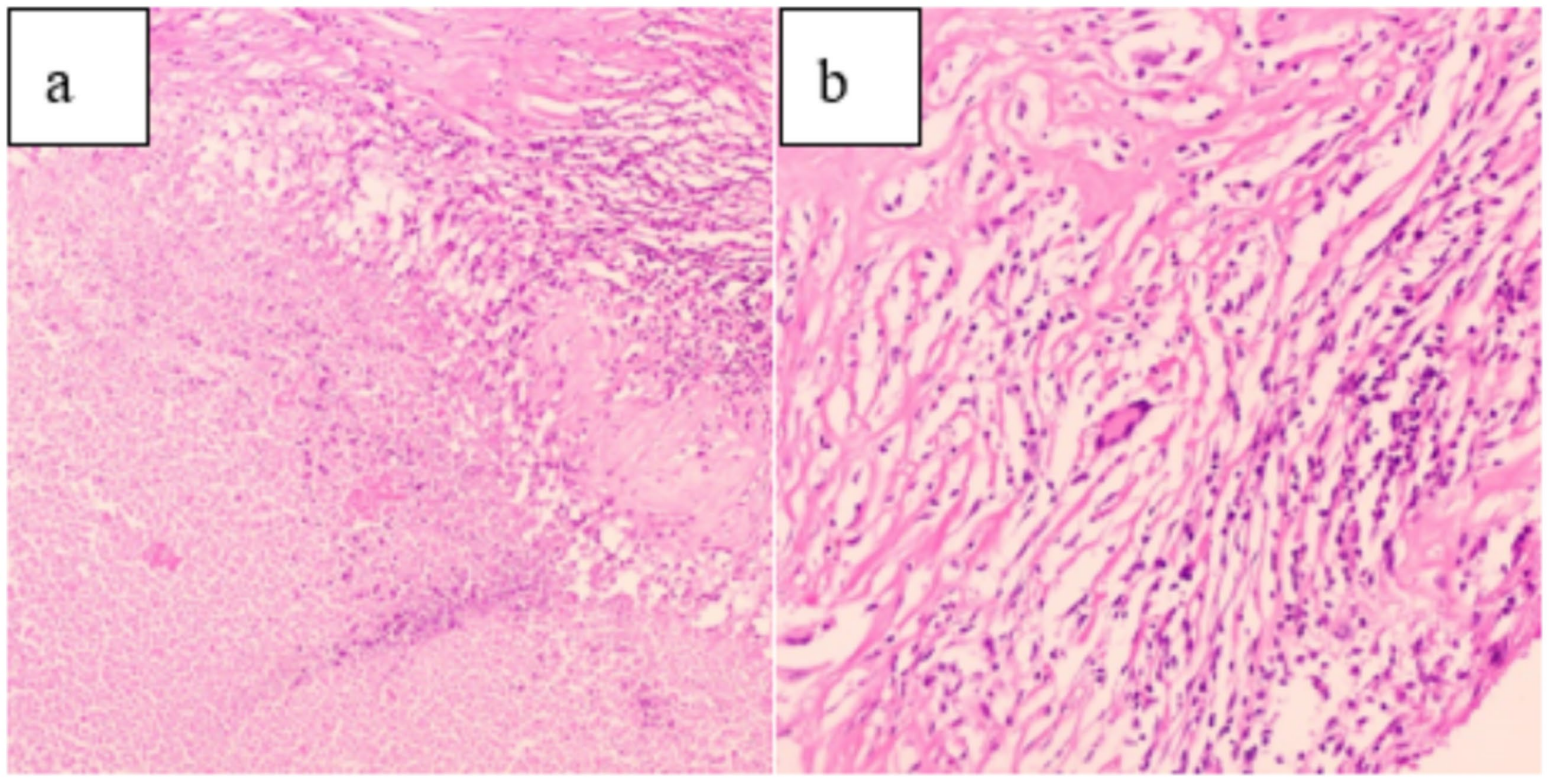
**(a)** (HE 10 × 10): Pathological examination showed caseous necrosis. **(b)** (HE 20 × 10): Central multinucleated giant cells and scattered peripheral lymphocyte infiltration.

After ruling out any surgical contraindications, laparoscopic radical resection of the left renal tumor was performed. Intraoperatively, the left kidney appeared significantly atrophic with an irregular surface. The kidney tissue showed severe damage, accompanied by a large amount of caseous purulent fluid suggestive of tuberculosis. A mass measuring approximately 30 × 30 mm was also identified. Pathological examination of the resected specimen revealed histological features consistent with both RCC and renal TB, including tumor cell infiltration (Figure 4), granuloma formation, and central caseous necrosis (Figure 5).

**Figure 6:**
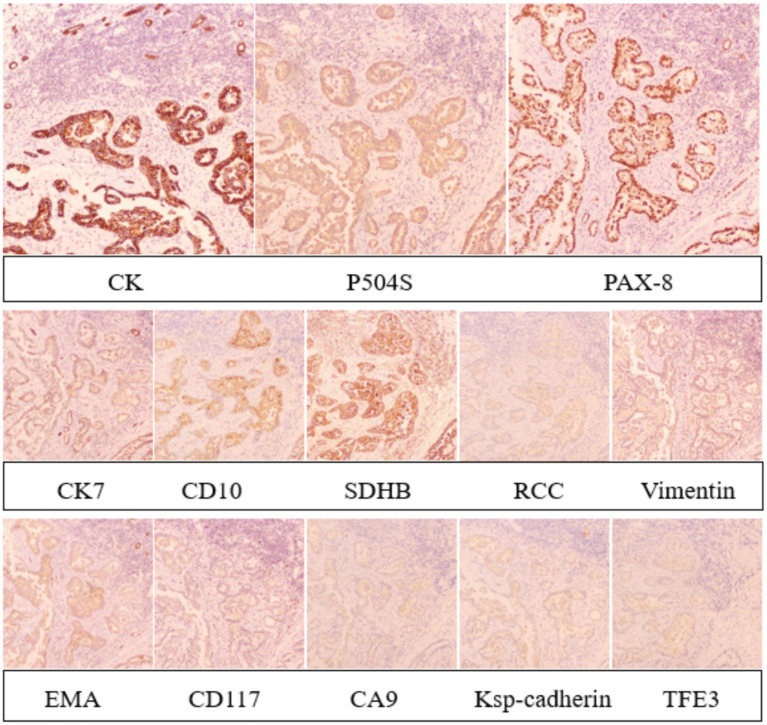
Immunohistochemical staining revealed strong positivity for CK, P504S, and PAX-8 in tumor cells, weak positivity for CK7, CD10, SDHB, RCC, and Vimentin, and negative staining for EMA, CD117, CA9, Ksp-cadherin, and TFE 3.

Immunohistochemical staining revealed strong positivity for CK, P504S, and PAX-8 in tumor cells, weak positivity for CK7, CD10, SDHB, RCC, and Vimentin, and negative staining for EMA, CD117, CA9, Ksp-cadherin, and TFE 3 (Figure 6). Molecular pathology confirmed the presence of TB DNA through PCR testing. The combined pathological and immunohistochemical findings led to the diagnosis of PRCC coexisting with TB at the left superior renal pole, along with perirenal fat infiltration by tumor cells.

**Figure 7:**
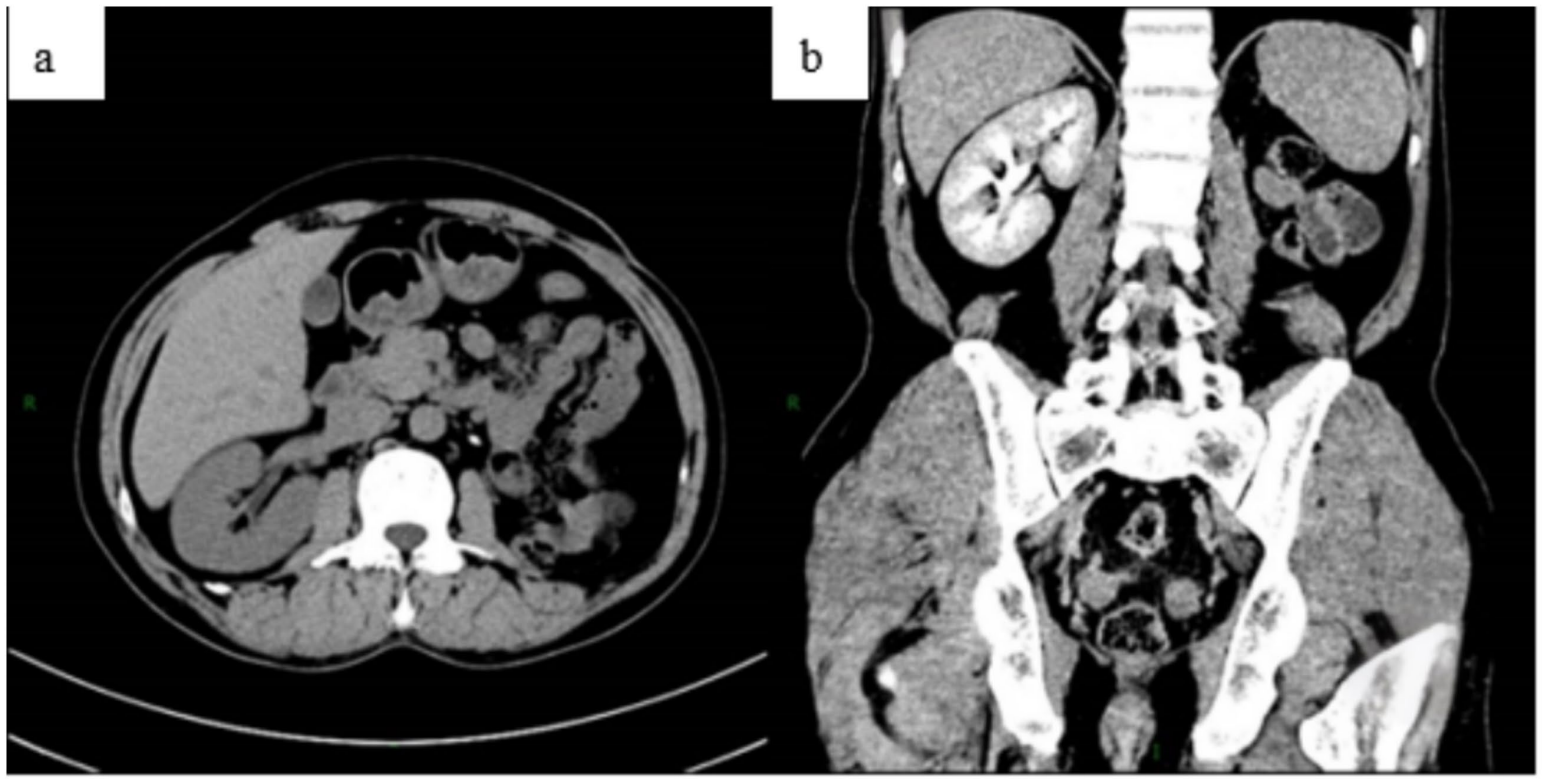
Abdominal CT was reviewed 3 months after surgery. The plain scan **(a)** and enhanced scan **(b)** showed no obvious abnormalities following the left nephrectomy.

The patient received anti-TB treatment with isoniazid, rifampicin, pyrazinamide, and ethambutol (The intensive phase involved a combination of four drugs for 2 months, followed by the consolidation phase with a combination of isoniazid and rifampicin for 4 months). Follow-up examinations, including repeat abdominal CT, demonstrated good postoperative recovery following left nephrectomy (Figure 7). During treatment with anti-TB drugs, the patient experienced no side effects, including decreased vision, peripheral neuritis, liver function impairment, or gastrointestinal discomfort. As of the latest manuscript revision, 11 months after surgery, the patient remains alive and in good health.

## Discussion

RCC is the 14th most common malignant tumor worldwide (6), with clear cell carcinoma representing about 80% of cases (1), making it the most prevalent histological subtype (8). Among the remaining 20%, papillary and chromophobe RCC are the most prevalent types (1, 9, 10). The new WHO classification of renal cancer subtypes represents a significant advancement in the personalized treatment of renal malignancies.

Renal TB has an insidious onset and lacks specific clinical manifestations. Common symptoms include hematuria, pyuria, dysuria, bladder irritation, fever, weight loss, and low back pain (11, 12). Pseudotumor-type renal TB is a very rare condition (13), characterized by imaging findings similar to renal tumors. It should be suspected when atypical renal masses are observed in patients from TB-endemic areas (14).

Tumors and TB predispose individuals to an autoimmune stress response within the host immune system, and malignancy is an independent risk factor for Mycobacterium TB infection (15). Tumors can cause TB reactivation, which in turn can promote tumor development, suggesting both a causal and symbiotic relationship (16). However, studies by Peyromaure and Redondo et al. (17, 18) suggest that the association between renal cancer and renal TB is exceedingly rare, with no causal or symbiotic relationship between the two.

Fibronectin, a component of the extracellular matrix, facilitates the attachment of live *MTB* to urinary epithelial cells. Bacillus Calmette-Guerin (BCG) bladder instillation immunotherapy for transitional cell carcinoma also relies on fibronectin-mediated mechanisms. The interaction between BCG, fibronectin, and tumor cells inhibits tumor cell motility and may contribute to TB infection (19). Regarding the concurrent occurrence of cancer and TB at the same site, some scholars suggest that chronic inflammatory mucosal damage triggers metaplasia and dysplasia, ultimately leading to tumor development (20, 21). Another hypothesis suggests that cancer invasion into dormant TB lesions activates *MTB* and triggers endogenous reinfection (22).

Lien et al. (23) found a strong association between urotuberculosis and urothelial carcinoma, identifying urotuberculosis as a high risk factor for urothelial carcinoma, but not for RCC (18). Renal cancer combined with renal TB is a rare and complex clinical condition, involving two diseases with distinct pathological mechanisms. The symptoms of the two diseases may be masked or confused with each other. For example, hematuria caused by renal TB may be mistaken for a clinical manifestation of renal cancer, and a mass from renal cancer may obscure the radiographic findings of renal TB.

Low back pain and hematuria are common clinical manifestations of both RCC and urinary TB. However, cases of RCC accompanied by ipsilateral renal TB are very rare. Fey et al. (24) first reported a case of ipsilateral renal cancer with renal TB in 1950. Similar cases were subsequently reported by Ducassou (25) and Kuriĭ (26).

In 2013, Mani et al. (27) reported a case of renal TB complicated by ipsilateral RCC. The patient presented with an incidental renal mass and underwent partial nephrectomy after evaluation. Postoperative pathological examination confirmed RCC, with multiple epithelioid cell granulomas observed within the tumor lobules, surrounded by lymphocytic infiltration. Central caseous necrosis and a few Langerhans giant cells were also identified within the granulomas. TB PCR confirmed the presence of Mycobacterium TB, thereby establishing the diagnosis of ipsilateral RCC complicated by renal TB.

However, previous case reports of RCC with concurrent renal TB have not documented PRCC. This case represents the first recorded instance of PRCC associated with ipsilateral renal TB.

The increased accuracy of abdominal CT in diagnosing RCC may contribute to the reduced mortality of patients. In patients with suspected RCC, enhanced CT of the chest, abdomen, and pelvis is required for staging, with additional MRI if necessary (6). There is a partial overlap between the imaging features of renal TB and those of benign and malignant renal tumors (28), which can contribute to clinical delays in diagnosis. Despite significant progress in CT and its high differential diagnostic value, achieving a qualitative diagnosis remains difficult (29). Ultrasound, CT, and MRI techniques have high sensitivity for detecting renal lesions, but none provides an accurate and reliable qualitative diagnosis. When the patient’s condition requires and clinicians have difficulty making a qualitative diagnosis, a renal biopsy should be considered (30).

After confirming the diagnosis of PRCC combined with renal TB, the preferred treatment method is anti-tubercular drug therapy combined with surgical resection of the affected kidney. Compared to open renal resection, laparoscopic radical resection of kidney tumors, which offers advantages such as less trauma, reduced intraoperative bleeding, faster postoperative recovery, and fewer postoperative complications (31), can be considered the first choice. Patients should return to the hospital for follow-up evaluations.

In future clinical scenarios, it is crucial to identify kidney masses early and make accurate qualitative diagnoses. Timely and systematic treatment can help preserve renal function and improve patient prognosis.

## Conclusion

PRCC with renal TB is a very rare and complex clinical condition that presents challenges in both diagnosis and treatment. Patients require multidisciplinary management with close monitoring of renal function and condition changes. In clinical practice, when patients present with symptoms such as low back pain and hematuria, and imaging examinations indicate a renal mass, we should consider the differential diagnosis of RCC and renal TB, and remain vigilant for the simultaneous occurrence of both diseases.

## Data Availability

The raw data supporting the conclusions of this article will be made available by the authors without undue reservation.
